# Conformational Plasticity of proNGF

**DOI:** 10.1371/journal.pone.0022615

**Published:** 2011-07-26

**Authors:** Francesca Paoletti, Francesca Malerba, Geoff Kelly, Sylvie Noinville, Doriano Lamba, Antonino Cattaneo, Annalisa Pastore

**Affiliations:** 1 European Brain Research Institute, Rome, Italy; 2 Scuola Normale Superiore, Pisa, Italy; 3 NMR Centre – MRC, London, United Kingdom; 4 Unité de Virologie and Immunologie Moléculaires, CR1-CNRS INRA, Jouy-en-Josas, France; 5 Istituto di Cristallografia, CNR, Trieste, Italy; 6 MRC National Institute for Medical Research, London, United Kingdom; University of Rome, Italy

## Abstract

Nerve Growth Factor is an essential protein that supports neuronal survival during development and influences neuronal function throughout adulthood, both in the central and peripheral nervous system. The unprocessed precursor of NGF, proNGF, seems to be endowed with biological functions distinct from those of the mature protein, such as chaperone-like activities and apoptotic and/or neurotrophic properties. We have previously suggested, based on Small Angle X-ray Scattering data, that recombinant murine proNGF has features typical of an intrinsically unfolded protein. Using complementary biophysical techniques, we show here new evidence that clarifies and widens this hypothesis through a detailed comparison of the structural properties of NGF and proNGF. Our data provide direct information about the dynamic properties of the pro-peptide and indicate that proNGF assumes in solution a compact globular conformation. The N-terminal pro-peptide extension influences the chemical environment of the mature protein and protects the protein from proteolytic digestion. Accordingly, we observe that unfolding of proNGF involves a two-steps mechanism. The distinct structural properties of proNGF as compared to NGF agree with and rationalise a different functional role of the precursor.

## Introduction

Nerve Growth Factor (NGF), the most prominent member of the neurotrophin family, is an essential protein that supports neuronal survival during development and influences neuronal function throughout adulthood, both in the central and peripheral nervous system [1]. NGF exerts its biological role by binding to two different receptors, TrkA, and p75^NTR^, respectively belonging to the tyrosine kinase and the Tumor Necrosis Factor receptor superfamilies [2], [3]. The structural determinants of NGF interactions with its receptors have been recently disclosed [4]–[6]. NGF is translated as a longer precursor known as pre-proNGF, which contains a signal peptide for protein secretion (pre-peptide) that is cleaved upon translocation into the endoplasmic reticulum to produce the precursor homo-dimer (proNGF) of *ca* 50 kDa. The pro-sequence (103 aminoacids) is then further processed in the trans-Golgi network by the furin protease at a highly conserved dibasic amino acid site and results in the release of the mature NGF dimer of *ca* 26 kDa [7]. Other proteases besides furin are also able to cleave the pro-peptide in the extracellular space [8], [9].

Initially, no specific biological role was attributed to the proNGF precursor, besides the demonstration that it regulates neurotrophin secretion. More recently, however, proNGF was found to be the predominant form of NGF in brain [10], to be produced at increased levels in Alzheimer's disease [10] and to induce p75^NTR^ dependent apoptosis in cultured neurons [8], all properties well distinct from those of the mature NGF [8]. Sortilin, a member of the family of the Vps10p-domain receptors, was discovered to be a specific receptor of proNGF. Sortilin binding to proNGF is essential for proNGF-induced neuronal cell death through p75^NTR^ [11]. A new model of neurotrophin activity is thus emerging which involves NGF, proNGF and the three receptors: TrkA (predominantly for NGF), sortilin (for proNGF) and p75^NTR^ (for both NGF and proNGF).

An essential step to investigate the distinct biological functions of proNGF *versus* NGF and rationalize how the pro-peptide extension may achieve functional specificity would be establishing the proNGF structure. The possibility of obtaining the mouse and the human pro-proteins in a functional recombinant form in different systems (*i.e*. *E.coli*, baculovirus, mammalian cells) [8], [12]-[15] has recently opened new avenues for carrying out more detailed studies of the precursor protein. Thanks to this possibility, the proNGF peptide was shown to act as an intramolecular chaperone and facilitate folding under oxidative conditions of recombinant human NGF expressed in *E. coli* [12], [16]. Conversely mature NGF was suggested to be strictly required for proNGF to be correctly folded and structurally organized for function [17]. Indications, albeit indirect, of interactions of the NGF moiety with the proNGF peptide region also came from *in vitro* chemical unfolding and indirect fluorescence studies of recombinant human proNGF that suggested an involvement of tryptophan 21 on the mature NGF [18]. However, while the X-ray crystallographic structure of NGF is available [19], structural determination of proNGF at high resolution remains elusive. A possible explanation for this lack of data is that the pro-peptide region seems to have features typical of an intrinsically unfolded region (IUR): preliminary structural studies of the isolated pro-peptide result in an unfolded species in agreement with sequence analysis [17], [20]. Further evidence in favor of a partial disorder of the pro-peptide was also provided by Small-Angle X-ray Scattering (SAXS) techniques which suggested the presence in solution of a conformational ensemble, with a prevalence of a compact arrangement of the pro-peptide extension in close contact with the mature NGF moiety [20], [21].

Several open questions remain however before we can fully understand the cellular role of proNGF: How is the structure and accessibility of NGF affected by the presence of the pro-peptide? What are the dynamical properties of the pro-peptide extension and how could they relate with their proposed intrinsically unfolded nature?

Here, we have used a set of complementary biophysical techniques which range from Fourier transform infrared (FT-IR), to circular dichroism (CD), differential scanning calorimetry (DSC) and nuclear magnetic resonance (NMR) analysis in the attempt of gaining a more detailed description of the tertiary structure of the pro-peptide in the context of the full-length protein. NMR in particular was used to gain a better picture of the dynamical properties of the two molecules. The view that emerges from our study is that the pro-peptide extension is rather rigid and behaves in solution as a compact globular domain. We also directly observe a strong influence of the pro-peptide on the NGF moiety. The new evidence sheds light onto the dynamical and structural features of the pro-NGF peptide and provides a new reference point for the structural role that pro-sequences have in essential biological functions.

## Results

### Secondary structure analysis of the pro-peptide of proNGF25

**Figure 1:**
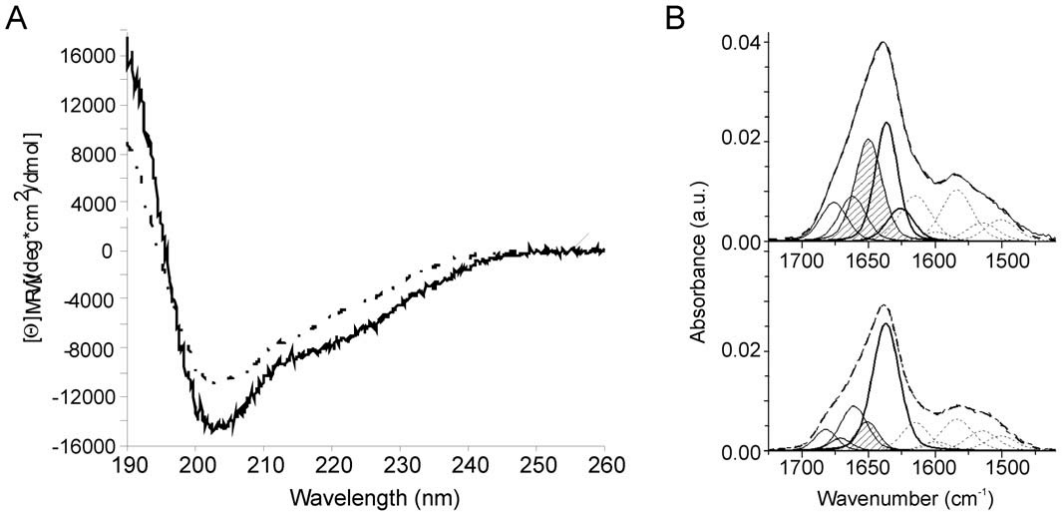
CD and FT-IR analysis of NGF and proNGF25. A) Far-UV CD spectra of NGF (black line) and proNGF25 (grey line) in 50 mM sodium phosphate. The difference curve proNGF25 subtracted NGF is represented by the dashed curve. B) Curve fittings of FT-IR spectra of proNGF25 (upper panel) and NGF (lower panel) in deuterated phosphate buffer at pD 7. The component bands present in the amide I' region are assigned to β-sheet (bold lines), to random domains (dashed line) and to turns (dot-dashed lines). The component bands in the 1600–1500 cm^-1^ region are attributed to side-chain and amide II contributions and are shown for eye guideline only. The sum of all these components is represented as a dashed line.

We first compared the overall secondary structure contents of recombinant murine proNGF (named hereafter proNGF25 to indicate the short form of the precursor protein used also in our previous studies [20]) and NGF in the attempt of getting some more quantitative estimate of the structural contribution of the pro-peptide. We used both CD and FT-IR spectroscopies, two highly complementary techniques. The far-UV CD spectroscopic data are in optimal agreement with those published for the human proteins (**Figure 1A**) [16]. The CD spectrum of proNGF25 has at 205 nm a much deeper and narrower band than that observed for NGF. Accordingly, the spectrum difference retains a minimum at 205 nm. The secondary structure contents estimated from these spectra are 38% extended, 12% turn, 43% random and 5% helix for NGF and 35% extended, 13% turn, 43% random and 7% helix for proNGF25. A predominant β-component for NGF is in agreement with the secondary structure calculated from the deposited crystal structure (PDB entry 1BET) [19].

To complement the CD data and obtain a more reliable secondary structure prediction, we performed FT-IR transmission measurements. The FT-IR spectra of NGF and proNGF25 recorded at 42 µM and 28 µM protein concentrations show for both proteins amide I' bands centred at 1638 cm^−1^ that are characteristic of proteins with a high content in β-structure (**Figure 1B** **)**. Decomposition of the NGF spectrum gives an estimate of 57% β-strands, 32% turns, 11% random, and no helical component (**Table 1**). The secondary structure composition of proNGF25 as determined by the same decomposition procedure is 42% β-strands, 26% turn and 32% random.

**Table 1:** Analysis of the Amide I' component bands and percentage of secondary structural elements from FT-IR spectral deconvolution of NGF and proNGF25.

Wavenumber (cm^−1^)	% Amide I'	Wavenumber (cm^−1^)	% Amide	Assignment to ν(CO) peptide groups
NGF		proNGF25		
1682	8			peptide CO in β-turns or random coil*
1671	5	1676	12	peptide CO in β-turns or random coild*
1661	19	1662	14	peptide CO in β-turns or random coil*
1651	11	1650	32	peptide CO in random coil
1637	56	1630	31	H-bonded CO in not-hydrated β-sheets*
1626	1	1626	11	H-bonded CO in hydrated β-sheets*

*The component at 1682 cm^−1^ could be a vibrational mode coupled to the mode appearing at 1637 cm^−1^ assigned to anti-parallel β-sheets.

These estimates are in reasonable agreement with each other, taking into account that the assignment of the IR component band at 1682 cm^−1^ can be also attributed to a coupling with the vibrational mode appearing at 1637 cm^−1^ which is assigned to anti-parallel β-sheets [22].

Taken together, these results suggest that the residues of the pro-peptide of proNGF25 do not increase significantly the random coil content of proNGF25 when compared to mature NGF and that therefore the pro-peptide contains at least some defined secondary structure.

### Optimization of expression, refolding and purification of proNGF25 in minimal medium for NMR spectroscopic studies

We used NMR to further analyse the structure of proNGF25 and obtain indications about the dynamics of the pro-peptide. This technique benefits from the use of ^15^N single and ^15^N/^13^C double labelled samples, which can be usually obtained by growing bacteria in minimal medium containing ^15^N ammonium sulphate and ^13^C glucose as the sole sources of nitrogen and carbon respectively. The first attempts to obtain labelled proNGF25 and NGF with traditional protocols [23] were however unsuccessful: we had very low yields, both at the level of expression and of refolding of the protein. To obtain suitable amounts of protein it became necessary to develop a new protocol, modified from Curtis–Fisk et al. [24], aimed at the optimization of the yields. The main differences with the previously described protocol are: i) a different composition of the growing media and induction time, and ii) a different refolding protocol (optimized for mouse proNGF [20]).

After refolding and purification, the typical final yield of purified proNGF25 was 1.5 mg/g of cell mass. NGF was obtained from proNGF25 by enzymatic cleavage and the purified NGF yield is around 0.5 mg/g of cell mass. These amounts were enough for the aims of the present studies although they would not easily allow full NMR spectral assignment that for proNGF25 would require triple ^15^N/^13^C/^2^H labelling, which usually results in a further drop in yields. The level of monodispersion of the proteins solutions was checked by DLS (data not shown). Overall, we found that while the unlabelled samples were always ≥90% monodispersed, labelled material had higher variability suggesting that growth in minimal medium introduces some level of stress. We therefore put extra care in always checking the proteins by DLS before NMR measurements.

### In solution proNGF25 behaves as a compact globular domain

**Figure 2:**
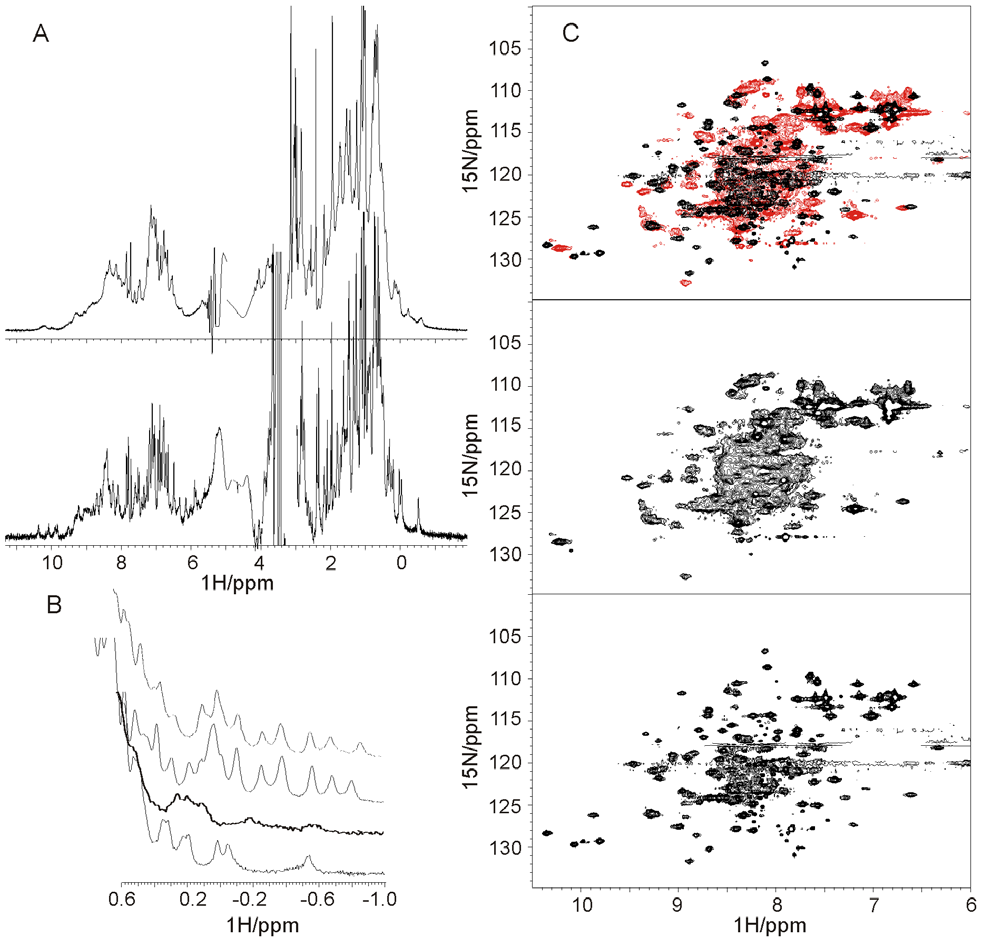
NMR spectral properties of NGF and proNGF25. A) Comparison of 1D spectra for NGF (lower spectrum) and proNGF25 (upper spectrum). B) Expansion of the high-field regions of the spectra shown in A) and comparison with the same regions of the spectra of ataxin-3 (35 kDa) and its N-terminal Josephin domain (20 kDa). The stacked plot shows from bottom up the spectra of NGF, proNGF25, Josephin and ataxin-3 respectively. C) 2D HSQC heteronuclear spectra of NGF (bottom), proNGF25 (middle) and overlay of the two (top) (NGF is shown in black, proNGF25 in red). The spectra were recorded on 0.4 mM protein concentrations, 25°C and at 800 MHz overnight.

The 1D NMR spectrum of unlabelled NGF, that is in excellent agreement with that of human NGF [25], has an intrinsic excellent dispersion and sharp linewidths indicating that the protein is well folded and not aggregated (**Figure 2A** **, lower panel**). The overall sharpness of the spectrum is compatible with the presence of a relatively small molecular weight species in solution, as it would be the expected NGF dimer (*ca* 26 kDa). In comparison, the spectrum of proNGF25 contains much broader resonances (**Figure 2A** **, upper panel**). This is in agreement with the bigger molecular weight of this protein (*ca* 50 kDa) which is also a dimer in solution [16].

A detailed comparison of the two spectra allows us to draw a number of solid conclusions even in the absence of spectral assignment. Particularly revealing are the high field regions. A set of proton resonances are observed in both spectra between −1 and 0.5 ppm (**Figure 2B** **, 1st and 2nd spectra from the top**). These are typical of aliphatic groups in close and persistent spatial proximity of aromatic groups (ring current shifts). The high field resonances of the proNGF25 spectrum have a pattern well distinct and definitely different from that observed for NGF, strongly suggesting that the pro-peptide adopts a well defined and persistent conformation in the context of the full-length protein. The behaviour observed for proNGF25 does not represent a general case for multidomain proteins. As a control, we considered the 1D spectrum of ataxin-3 and of its isolated N-terminal domain Josephin [26]. The Josephin domain of ataxin-3 is known to be the only globular and structured region of the protein, whereas the C-terminus is flexible [26]. Accordingly, we observe that the spectra of the full-length protein and of the domain are roughly additive and that the presence of the unstructured C-terminus does not significantly influence the spectrum of Josephin (**Figure 2B** **, 3^rd^ and 4^th^ spectra from the top**).

These results were further supported by 2D HSQC heteronuclear spectra (**Figure 2C**). Once again, the HSQC of ^15^N labelled NGF is relatively sharp and well dispersed, whereas that of ^15^N labelled proNGF25 contains very broad resonances clustered together. There is no evidence that could suggest the presence of double species which could correspond to an asymmetric dimer either for NGF or from proNGF25. This is for instance clear for the resonances of the C-terminal residue (likely the resonance at 7.9 ppm and 128 ppm) and of the indole protons of the three tryptophans of proNGF25 (likely the peaks around 10 ppm and 130 ppm). This behaviour is well in agreement with the occurrence of a two fold symmetry that relates NGF monomers, as observed in the crystal structures 1BET and 1BTG [19], [27] and that might also apply to the pro-peptide extension of proNGF25.

Interestingly, the resonances of the tryptophan indole protons (Trp21, Trp76, Trp99 for NGF and Trp −85, Trp21, Trp76, Trp99 for proNGF25) are well visible in the NGF spectrum whereas in the proNGF25 spectrum there seem to be at least two different populations, one represented by a resonance at 10.4 ppm and 128 ppm, the other represented by two smaller peaks present in all the spectra recorded from independent batches of protein. These peaks could be indicative of the co-presence of two species with distinct dynamical properties.

**Figure 3:**
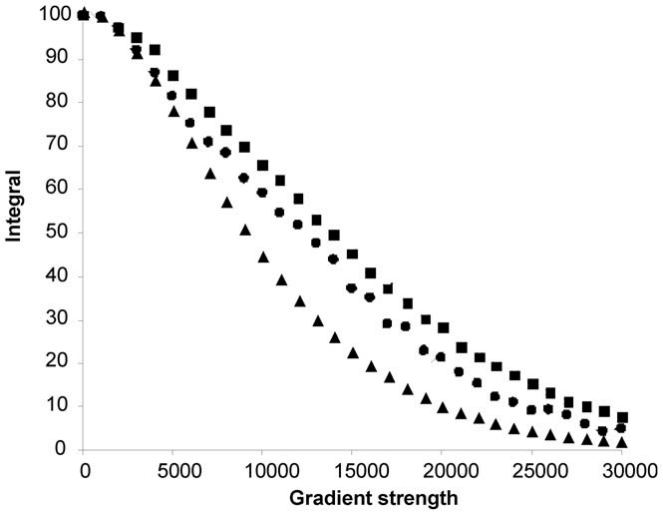
NMR diffusion data. The plot reports the integral of an envelope of signals (y-axis) against the applied gradient strength (x-axis). The symbols correspond to the IscS dimer (grey squares), proNGF25 (black squares), DHFR (grey diamonds), NGF (black circles) and the NusA KH domain (black triangles). For each protein, the peak integrals were plot against the applied gradient strength.

To get a semi-quantitative indication of the correlation time of proNGF25 and the degree of compactness of the molecule, we compared the diffusion of this protein as measured by NMR with that of NGF and of other well characterised model proteins known to be monodispersed in solution and to form compact globular structures without major shape anisotropy. This method has been shown to be efficient in providing indications on the size as well as on the shape of proteins [28]. We observed that the diffusion of the proteins used for comparison correlates well with their expected molecular weights: plots of resonance intensities as a function of gradient field strength give sigmoidal dependence which can be translated into diffusion constants. On the same calibration curve, the proNGF25 curve is found between the IscS dimer (90 kDa) and DHFR (20 kDa), which is consistent with a molecular weight of 50 kDa of the dimer (**Figure 3**). The curve of NGF (26 kDa) appears below together with smaller molecular weight markers. These results not only confirm that the protein is monodispersed in solution, but also that it behaves as a globular protein, with no major features of an elongated shape or of unfolded flexible regions in agreement with the described compact model of proNGF25 obtained by SAXS in solution [20].

### Intramolecular interactions detected by in tube proteolytic digestion

Digestion of proNGF25 in the NMR tube was carried out to find further evidence in favour of structural intramolecular interactions between the pro-peptide and the mature NGF. Different proteases are known to cleave proNGF25 (**Figure S1**). We first used the unspecific protease trypsin, which, as we and others have demonstrated, cleaves the more accessible pro-peptide fragmenting it but leaves NGF undigested [16], [20]. We adjusted the conditions (trypsin∶proNGF25 ratios, temperature and buffer composition) to have a reasonably slow kinetics of proteolysis which could be comfortably followed by NMR. We found optimal conditions at 1∶10000 trypsin∶proNGF25 ratios, at 25°C.

When following the reaction overnight, we could observe a clear transition during the kinetics from the proNGF25 to the NGF 1D ^1^H spectrum (**Figure S2**). As the reaction proceeds, the chemical environment evolves towards that of the isolated mature NGF. At the end of the reaction no proNGF25 is left uncleaved as judged by sodium dodecyl sulphate polyacrylamide gel electrophoresis (SDS-PAGE) (**Figure S3**). The identity of the pro-peptide fragments generated by the trypsin cleavage was confirmed by mass spectrometry which revealed no fragment arising from mature NGF (data not shown). This observation is in agreement with a shielding effect of the NGF moiety by the pro-peptide, as also reported by Kliemannel et al. [18].

**Figure 4:**
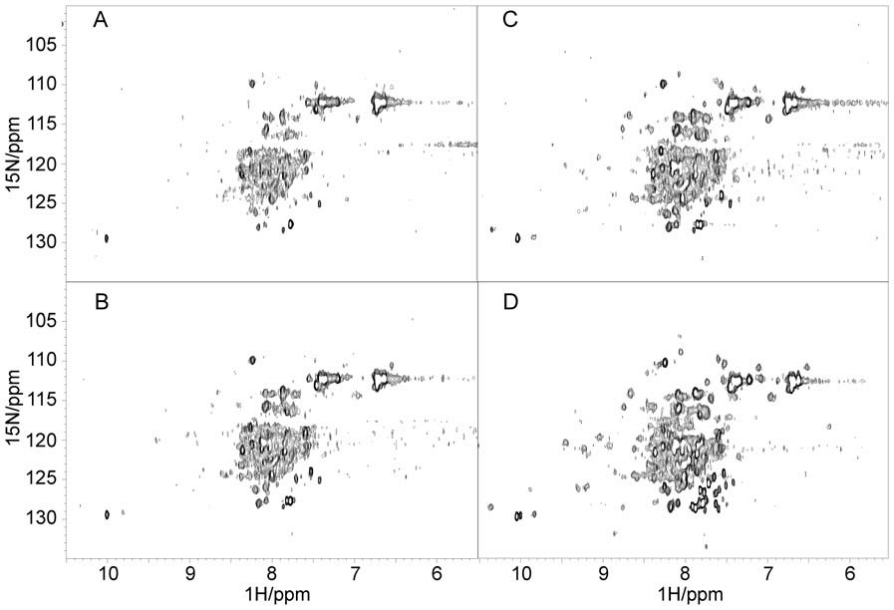
Enzymatic cleavage of the pro-peptide as followed by NMR. A) 2D HSQC heteronuclear spectrum of proNGF25 before addition of furin, B) immediately after furin addition, C) at completion of the cleavage reaction and D) after further addition of trypsin. The spectra were recorded at 25°C and 600 MHz. The initial sample was 0.1 mM, double labelled and less monodispersed than the one shown in Figure 2C (top and middle panels).

We then used the specific furin protease which cleaves proNGF25 at a specific site (consensus site RSKR, position 118–121, Uniprot entry P01139) and releases the intact pro-peptide as well as the mature NGF, as previously shown [20]. The reaction was followed over 24 hours by more informative 2D ^15^N HSQC spectra. After this time, SDS-PAGE confirmed completion of the reaction, with no undigested proNGF25 remained (**Figure S3**). Comparison of the spectra before and immediately after proteolytic cleavage (**Figure 4A,B**) shows a clear change of the spectrum. The peak at 7.9 ppm and 139 ppm which should correspond to the C-terminus, for instance, appears to be doubled, indicating the appearance of a second species. The tryptophan region becomes more populated, as already observed in the NGF HSQC spectrum. Comparison of the final HSQC with the one of NGF alone shows, however, that the well dispersed HSQC spectrum of NGF could not be resumed completely (**Figures 2C** **and** **4C**), suggesting the presence of residual interactions between the pro-peptide domain and NGF, with a consequent shielding effect of the NGF moiety.

To confirm this hypothesis, we further added trypsin to completely hydrolyze the pro-peptide domain. The reaction was slower than expected. This was presumably because it was carried out in HEPES, a buffer optimal for furin, but not for trypsin [20]. After 24 h of digestion, the HSQC spectrum resumes most of the features of the NGF spectrum (**Figure 4D**) although in the presence of enhanced protein degradation as assessed by the appearance of several small resonances. These results confirm a role of the pro-peptide in influencing the spectrum of the NGF moiety and further support an intramolecular interactions between the NGF and the pro-peptide.

### The pro-peptide stabilizes unfolding of NGF

**Figure 5:**
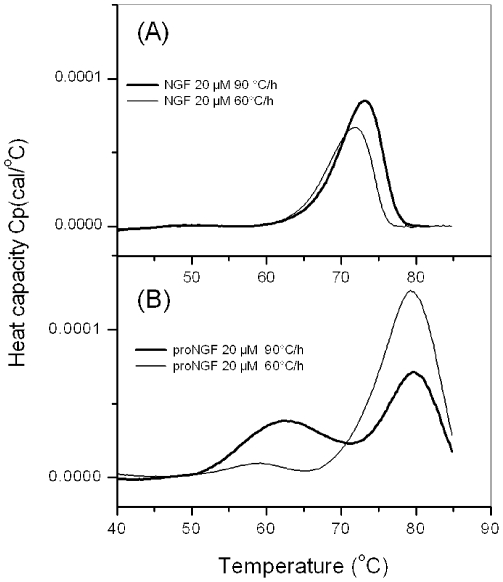
DSC thermograms of proNGF25 and NGF. DSC thermograms were recorded for solutions in a saline phosphate buffer at pH 7. A) NGF at 20 µM and heating rates of 90°C/h (bold line) and 60°C/h (thin line), B) proNGF25 at 20 µM and heating rates 90°C/h (bold line) and 60°C/h (thin line).

While seeking further confirmation about the effects of an interaction between the pro-peptide and the NGF domain, we compared the biophysical stabilities of NGF and proNGF25 by DSC bearing in mind that both proteins are obliged dimers which cannot exist as monomers in a native form. Under physiologic-like conditions (pH 7, 25°C and 20 µM protein concentration), denaturation is irreversible. This must be caused by aggregation of the unfolded state as revealed by the turbidity of the samples after each heating scan. Accordingly, the DSC thermograms are scan rate dependent for both proteins but have otherwise quite different features (**Figure 5**). At the same protein concentration, the DSC thermograms of NGF show only a single transition at 73°C, which could correspond to a two-state/dissociation process in which only the native dimeric state and the unfolded state are populated in solution. Conversely, the DSC thermograms of proNGF25 present two transitions, the first one around 60–65°C, the second at 79°C. Aggregation and precipitation starts at 81°C for NGF, whereas the one of proNGF25 occurs at 85°C.

These results indicate that the pro-peptide stabilizes the mature moiety, both in terms of unfolding temperature and of tendency to aggregate. Further interpretation of the data is currently made difficult because of the irreversible nature of the transitions.

## Discussion

Despite its undoubted biological interest, a 3D structural description of proNGF remains elusive, while the structure of mature NGF has been available for several years [19]. This lack of information together with difficulties encountered in crystallizing proNGF have been ascribed to the dynamic properties of the pro-peptide, which has been suggested to have features of an intrinsically unstructured region (IUR) [20]: bioinformatics analysis predicts this region as a IUR and SAXS data could be fit better under the assumption of a conformational ensemble in solution. Addition of ammonium sulphate also seemed to result in an overall more compact structure. The concept of IUR or intrinsically unfolded proteins (IUPs) was introduced a few years ago and has rapidly gained large popularity amongst the protein structure community [29], [30]. It designates proteins or protein regions devoid of a well defined tertiary structure. Some of them are flexible linkers necessary to allow other portions of the protein to move and interact. Others are able to acquire a structure and become rigid only upon interaction with a substrate, according to an induced-fit mechanism or to what has been recently generalized in the concept of ‘conformational fuzziness’ [31]. It is now widely accepted that a large portion of proteins in all genomes are IUPs or possess IURs, which thus play important roles in several different cellular pathways.

Here, we have used different complementary techniques, which range from NMR, to CD, FT-IR and DSC, to provide a more detailed picture of the structural behaviour of proNGF25. Both FT-IR and far-UV CD data show that the pro-peptide of proNGF25 does not contribute to a significant increase of random coil content of proNGF25 as compared to the mainly β-folded NGF, suggesting that the pro-peptide must contain at least some regular secondary structure, when observed in the context of the full-length protein. This is at variance with CD data carried out on the isolated pro-peptide which has all the features of a random coil [17] and implies that, although not adopting a unique conformation, the structure of the pro-peptide is strongly influenced by the NGF moiety. These conclusions are supported by other even more direct pieces of evidence. We conclusively observe that proNGF25 assumes in solution a compact globular conformation, as supported by mono- and bi-dimensional NMR spectra and by diffusion measurements. Our NMR data also suggest that the pro-peptide has no extended flexible or unstructured regions as those observed in other proteins which contain extended IURs, such as ataxin-3 [26], prions [32] or p53 [33], whose N- or C-terminal regions form highly flexible conformational ensembles that cover a large portion of the available conformational space. The pro-peptide has significant influence on the spectrum of mature NGF which is obtained back not by simple furin cleavage but only upon complete hydrolysis of the pro-peptide. Finally, we observe that the presence of the pro-peptide stabilizes the mature region as suggested by DSC.

Taken together our results all point towards a distinct structural and dynamical behaviour of NGF and proNGF in excellent agreement with previous data based on fluorescence spectroscopy and H/D exchange measurements which have also proposed an influence of the pro-peptide on NGF [18], [20]. Among other aspects, our conclusions could explain the different affinities of NGF and proNGF for their common receptors, TrkA and p75NTR, not only through an increased steric hindrance introduced by the pro-peptide, but also through a direct induced change in the mature NGF structure [34].

**Figure 6:**
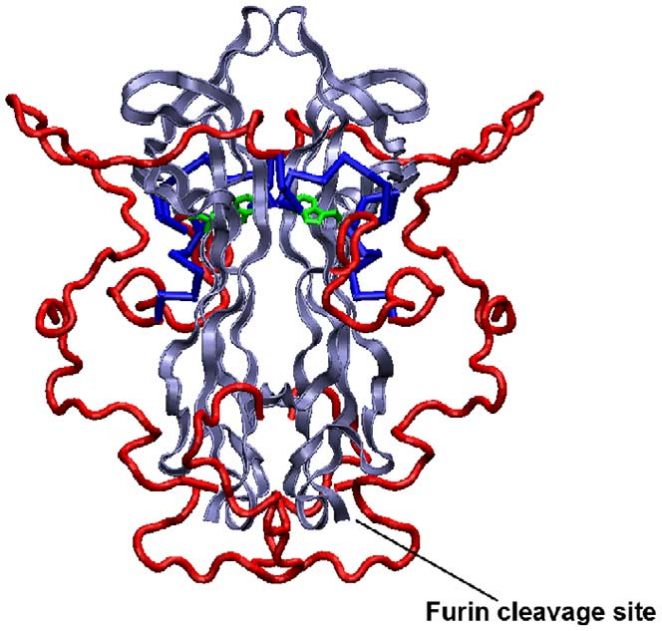
Model of proNGF25. The model was built according to previous SAXS data [20]. Mature NGF is shown in blue, the pro-peptide domain in red. The side chain of NGF Trp21 is shown in green. This residue was suggested to take part to the interaction of the NGF moiety with the pro-peptide [18]. The position of the furin cleavage site is also indicated.

We cannot yet map the exact interaction sites on both moieties as this would require NMR spectral assignment: the intrinsic poor spectral dispersion, the dynamical properties of proNGF25 and its size would require deuteration whereas we have low expression yields and misbehaviour already at the level of ^15^N labelled samples. While trying to circumvent the problem with alternative protein expression and labelling strategies, the data provided here suggest a new way to consider the structural role of the pro-peptide and possibly a new family of IUPs. We suggest a model in which proNGF follows what we could name a ‘conflicting fit’ mechanism as opposed to the more commonly spread ‘induced fit’. In the latter, an intrinsically unfolded chain becomes rigid and structured only upon interaction with a partner. With proNGF, we do not, on the contrary, observe obvious elements of flexibility and the pro-peptide seems to contain at least some secondary structure, thus not classifying directly as a bona fide IUP. However, the different conformations hinted by SAXS data [20] would agree well with a model in which, in the absence of inter-molecular partners, the relatively hydrophobic pro-peptide does not freely fluctuate in solution but collapses onto the mature NGF moiety in a “folded arms”-like state (**Figure 6**), as we and others have observed in terms of protection of the NGF moiety by the pro-peptide. The presence of an inter-molecular binding partner could however be sufficient for promoting a conformational change and stabilizing a different more-elongated conformation. In this conformation, the two arms formed by the pro-peptide would be ideally suited for establishing tight and specific interactions with the partner.

Further studies and determination of the proNGF structure in a complex with one or more of its cellular interacting partners is now required to conclusively validate this hypothesis and to finally gain a complete description of the proNGF structure at atomic resolution, in order to unable a full molecular understanding of its biological activity.

## Materials and Methods

### Optimization of expression, refolding and purification of proNGF25 in minimal medium

We inoculated 300 ml of Luria broth (LB) medium added with Ampicillin from a glycerol stock of BL21(DE3) *E. coli* transformed with the plasmid pET11a containing the gene of murine proNGF25 [20]. Cells were grown overnight at 37°C with shaking at 250 rpm until an OD_600_ of about 2.5. This step of growth in a complete rich medium was performed to obtain a considerable cell mass before induction in minimal medium. Cells were harvested at 1500 *g* for 5 min. The pellets were first washed and then resuspended in 250 mL of M9 minimal medium containing 42 mM NaHPO_4_, 22 mM KH_2_PO_4_, 8.5 mM NaCl, 2 mM MgSO_4_, 0.1 mM CaCl_2_, 1 g/L ^15^N-enriched ammonium sulphate and 3 g/L of either ^12^C or ^13^C glucose for single or double labelling. Thiamine and D-Biotine were added at 10 µM and 1 µg/mL, respectively. After 1 h of cell growth, protein expression was induced by 1 mM addition of isopropyl-β-thio-galactoside (IPTG). The proNGF25 production was continued for 5 h at 37°C at 250 rpm shaking. The cell pellet was harvested by centrifugation at 10000 *g* for 20 minutes.

### Refolding from inclusion bodies

proNGF25 forms insoluble aggregates inside the cells when over-expressed in *E. coli*. The procedure of solubilization and refolding of protein from inclusion bodies was carried out according to Rattenholl et al. [12], [16]. For the production of single and double labelled proNGF25 it was necessary to strictly maintain the following ratios: 2.5 mL of solubilization buffer/g of inclusion bodies; 50 mL of refolding buffer for 1 mL of solubilized inclusion bodies. NGF was obtained from an enzymatic proteolytic cleavage of proNGF25 by trypsin [20].

### Dynamic Light Scattering experiments

Dynamic light scattering (DLS) measurements of proNGF25 and NGF were performed to check the degree of monodisperion of the samples using 1 mg/mL concentrations of protein in 50 mM sodium phosphate, pH 7, at 25°C on a Viscotek 802 DLS. Samples were centrifuged at 12000 g for 10′ to remove precipitate or dust particles and then injected in a 12 µl quartz microCUVETTE (Wyatt Technology). Data were analyzed using the Software OmniSize.

### CD analysis

CD measurements were carried out with a JASCO J-810 circular dichroism instrument at 20°C in 50 mM sodium phosphate, pH 7.0. Far-UV CD (190-250 nm) spectra were recorded at a protein concentration of 0.5–1.0 mg/mL in a 0.02 cm cell (Hellma), averaged over 10 accumulations (with an acquisition time of 2 s). Spectra were buffer corrected. Mean ellipticity values were calculated as previously reported [35]. The secondary structure prediction was performed with DICHROWEB [36].

### Secondary structures from FT-IR analysis

Transmission IR spectra were recorded on a Nicolet 850 spectrometer equipped with a MCT detector. Resolution was set at 4 cm^−1^ using a boxcar apodization. The spectrometer was continuously purged with dry air. The IR cell was thermostated at 20°C. Absorbance spectra of solutions were obtained using CaF_2_ cells with a 50 µm spacer. We obtained deuterated NGF and proNGF25 solutions at the respective monomer concentration of 42 µM and 28 µM in deuterated phosphate buffer, by centrifugation of the NGF and proNGF25 solutions on deuterated Sephadex columns. Deuterium oxide (D_2_O) was obtained from Eurisotop (France). The extent of secondary structure was estimated from the amide I' band in the spectral range 1600–1700 cm^−1^ [22]. Second derivative analyses of IR spectra gave the fixed wavenumbers for each protein (Table 1) and the assignments were deduced from previous works [22], [37], [38]. The spectral decomposition for each protein was performed as previously described procedure [37].

### DSC experiments

DSC measurements were performed on a VP-DSC Microcalorimeter (Microcal). The temperature was increased from 20°C to 95°C. The scan rate was either 90°C/h or 60°C/h. The protein sample was dialyzed against the same buffer (50 mM phosphate, 150 mM NaCl pH 7) to minimize the difference between the reference buffer and the sample solvent. Thermograms were recorded at NGF or proNGF25 monomeric concentrations of 20 µM for comparison and repeated at 40 µM for NGF. The data were analyzed with Origin 7.0 software (Microcal).

### NMR experiments

NMR experiments were typically carried out on samples 0.1–0.3 mM in phosphate buffer at pH 7 at 25°C and a VARIAN INOVA spectrometer working at a 600 MHz proton frequency. Water suppression was obtained by the WATERGATE sequence. Diffusion measurements were performed using the PFG-LED pulse sequence [28], and calibrated with model system proteins known to be monodisperse in solution under the conditions used: the desulphurase IscS dimer (90 kDa) [39], dihydrofolate reductase (DHFR, 16 kDa), the proNGF25 (50 kDa) and NGF (26 kDa) dimers and an extended KH domain from NusA (6 kDa).

### *In vitro* Limited Proteolysis of proNGF25

Limited proteolysis experiments on proNGF25 with trypsin in the NMR tube were initially carried out as previously described [20]. However, since the kinetics were too fast to follow the process by NMR, the protocol was modified: proNGF25 (1 mg) was incubated in the NMR tube with 0.1 µg of trypsin in a total volume of 700 µL buffer (50 mM Tris-HCl, at pH 8, 2 mM CaCl_2_). The reaction was followed by recording short interleaved 1D and HSQC experiments over 24 h. Proteolysis with furin was carried out by first dialysing the proNGF25 sample against 100 mM HEPES at pH 7.4. The reaction was started in the NMR tube using 1 mg proNGF25 and 12 U of furin, in a total reaction volume of 700 µL buffer (100 mM HEPES at pH 7.4, 1 mM CaCl_2_). After 24 h, 400 ng of trypsin were added and the reaction was followed for additional 24 h. Both measurements were carried out at 25°C.

## Supporting Information

Figure S1 **Sequence of proNGF25.** The pro-peptide is indicated in italics, the NGF moiety in bold. The furin cleavage site is indicated in light blue. Trypsin cleavage sites are highlighted in red.
(TIF)

Figure S2 **Effect of enzymatic cleavage on the 1D spectrum of proNGF25.** A) Comparison of the proNGF25 proton spectrum (top trace) recorded at 25°C and 600 MHz with that of the same sample after 24 hours of trypsin cleavage. B) Close up of the high field regions of the time course of trypsin cleavage recorded at ∼4 h intervals.
(TIF)

Figure S3 **SDS-PAGE of the digestion of proNGF25 with both trypsin and furin.** The blue and red arrows mark NGF and proNGF25 respectively. The samples were analyzed at the indicated times.
(TIF)
