# Supplementary figures and images for: Conformational Plasticity of proNGF
Source: PLoS One. 2011 Jul 26;6(7):e22615. doi: 10.1371/journal.pone.0022615 (PMC3144226; doi:10.1371/journal.pone.0022615)

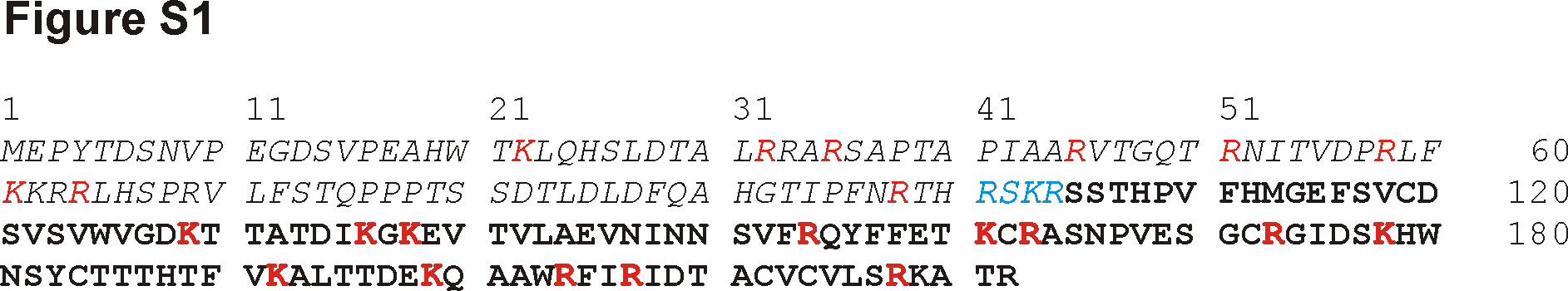

Supplement: Figure S1 — Sequence of proNGF25. The pro-peptide is indicated in italics, the NGF moiety in bold. The furin cleavage site is indicated in light blue. Trypsin cleavage sites are highlighted in red. (TIF) [file pone.0022615.s001.tif]

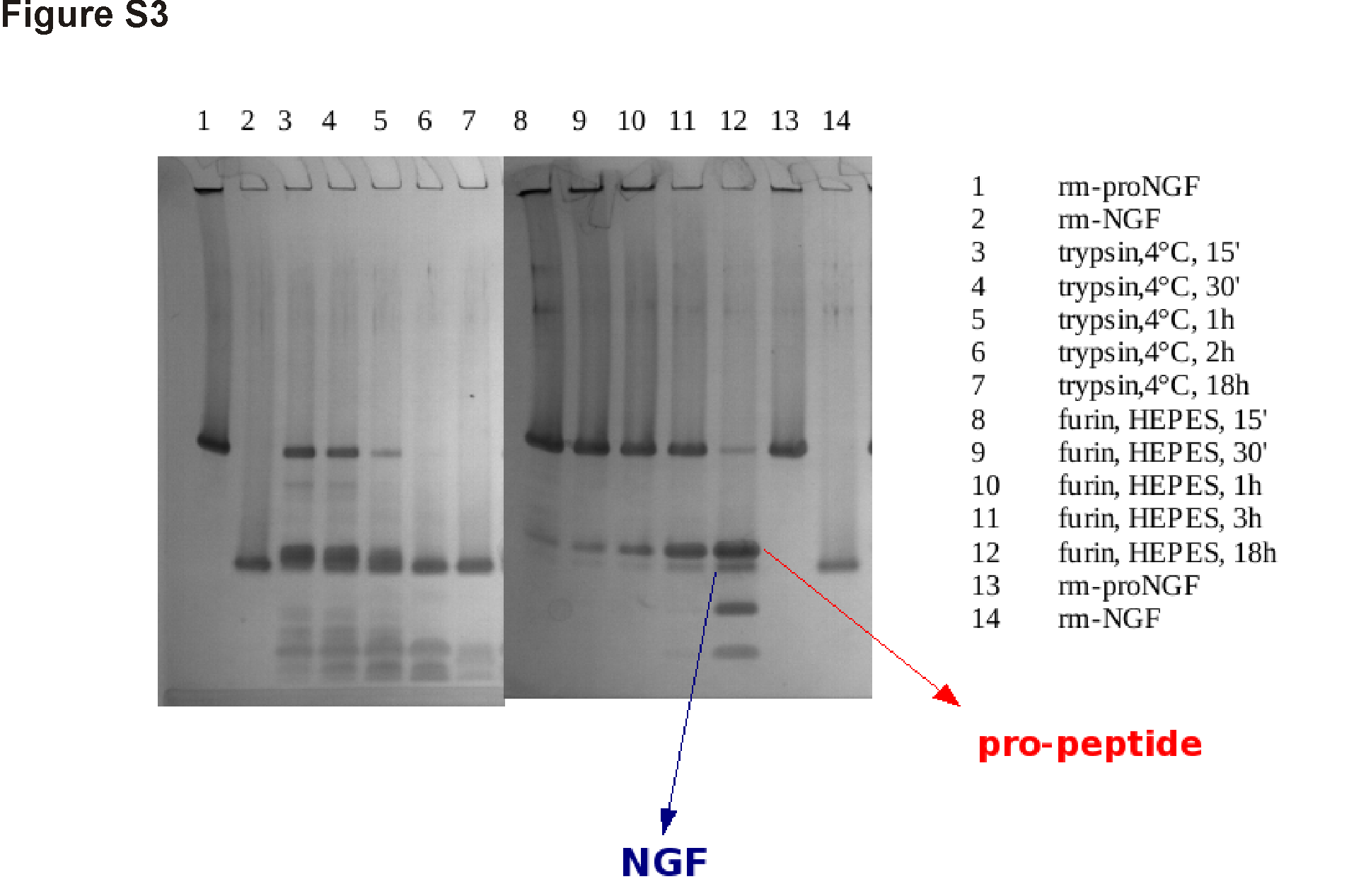

Supplement: Figure S3 — SDS-PAGE of the digestion of proNGF25 with both trypsin and furin. The blue and red arrows mark NGF and proNGF25 respectively. The samples were analyzed at the indicated times. (TIF) [file pone.0022615.s003.tif]
